# Comparative RNA-Sequencing Analysis Benefits a Pediatric Patient With Relapsed Cancer

**DOI:** 10.1200/PO.17.00198

**Published:** 2018-04-19

**Authors:** Yulia Newton, S. Rod Rassekh, Rebecca J. Deyell, Yaoqing Shen, Martin R. Jones, Chris Dunham, Stephen Yip, Sreeja Leelakumari, Jingchun Zhu, Duncan McColl, Teresa Swatloski, Sofie R. Salama, Tony Ng, Glenda Hendson, Anna F. Lee, Yussanne Ma, Richard Moore, Andrew J. Mungall, David Haussler, Joshua M. Stuart, Colleen Jantzen, Janessa Laskin, Steven J.M. Jones, Marco A. Marra, Olena Morozova

**Affiliations:** **Yulia Newton**, **Jingchun Zhu**, **Duncan McColl**, **Teresa Swatloski**, **Sofie Salama**, **David Haussler**, **Joshua M. Stuart**, and **Olena Morozova**, University of California Santa Cruz Genomics Institute; **Sofie Salama** and **David Haussler**, Howard Hughes Medical Institute, University of California, Santa Cruz, CA; **S. Rod Rassekh**, **Rebecca Deyell**, **Chris Dunham**, **Glenda Hendson**, **Anna F. Lee**, and **Colleen Jantzen**, British Columbia Children’s Hospital and British Columbia Children's Hospital Research Institute; **Stephen Yip**, **Tony Ng**, and **Janessa Laskin**, British Columbia Cancer; and **Yaoqing Shen**, **Martin Jones**, **Sreeja Leelakumari**, **Yussanne Ma**, **Richard Moore**, **Andrew J. Mungall**, **Steven J.M. Jones**, and **Marco A. Marra**, Canada's Michael Smith Genome Sciences Centre, British Columbia Cancer, Vancouver, British Columbia, Canada.

Clinical detection of sequence and structural variants in known cancer genes points to viable treatment options for a minority of children with cancer.^1^ To increase the number of children who benefit from genomic profiling, gene expression information must be considered alongside mutations.^2,3^ Although high expression has been used to nominate drug targets for pediatric cancers,^4,5^ its utility has not been evaluated in a systematic way.^6^ We describe a child with a rare sarcoma that was profiled with whole-genome and RNA sequencing (RNA-Seq) techniques. Although the tumor did not harbor DNA mutations targetable by available therapies, incorporation of gene expression information derived from RNA-Seq analysis led to a therapy that produced a significant clinical response. We use this case to describe a framework for inclusion of gene expression into the clinical genomic evaluation of pediatric tumors.

## CASE SUMMARY

**Fig 1.**
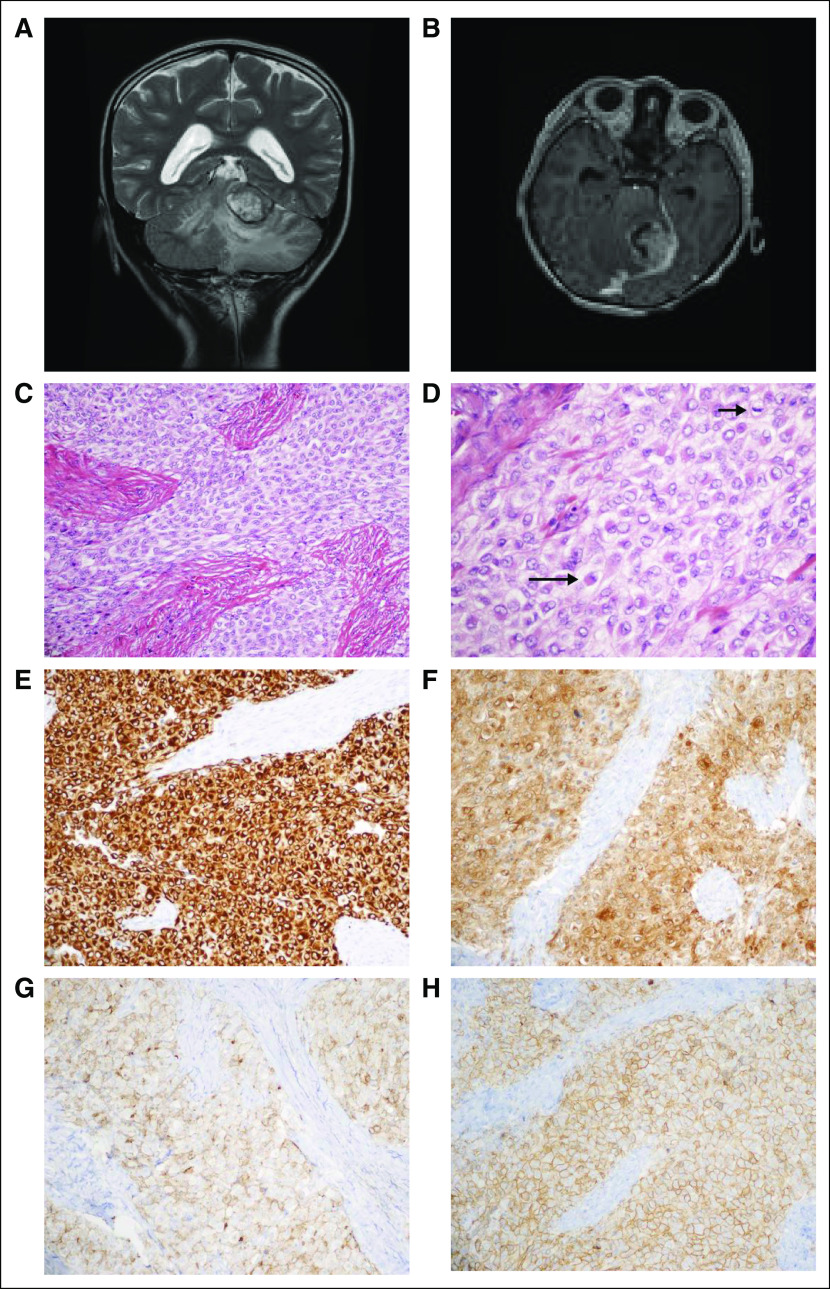
Case clinical information. (A) Preoperative magnetic resonance imaging (MRI). T2-weighted coronal sequence revealed a large, primarily hyperintense tumor that arises from the ventral aspect of the left tentorium, invaginating into the superior aspect of the cerebellum and causing diffuse edema therein. (B) Preoperative MRI. T1-weighted axial sequence with gadolinium revealed strong enhancement. (C) Routine tumor histology. Hematoxylin and eosin (H&E)–stained representative section revealed a combination of epithelioid and spindled tumor cells among thick fibrous bands (× 200 magnification). (D) Routine tumor histology. Higher magnification depicts epithelioid tumor cells (eg, long arrow) and a mitotic figure (short arrow); many of the tumor cells exhibit somewhat vacuolated cytoplasm (H&E; × 400 magnification). (E-H) Tumor immunohistochemistry. Strong diffuse cytoplasmic immunostaining is exhibited for (E) desmin and (F) neuron-specific enolase (NSE). Diffuse membranous immunostaining is appreciated for (G) epithelial membrane antigen (EMA), and (H) CD99 (all photomicrographs taken at × 200 magnification).

Patient 1 was diagnosed at 8 years of age with a left tentorial-based CNS sarcoma after a 2-week history of nausea, lethargy, and diplopia. Clinical workup confirmed that the tumor was primary to the brain (Figs 1A and 1B). Histology revealed a mitotically active, epithelioid-to-spindled cell tumor in patternless sheets, interrupted by thick fibrous bands and foci of necrosis (Figs 1C to 1D). Immunohistochemistry revealed diffuse positivity for vimentin, desmin, neuron-specific enolase, epithelial membrane antigen, and CD99 (Figs 1E to 1H). Focal immunohistochemical positivity was observed for pan-cytokeratin (AE1/AE3) and synaptophysin. The tumor was negative for glial fibrillary acidic protein (GFAP), Wilms tumor 1 (WT1), myo-D1, myogenin, smooth muscle actin, nonphosphorylated and phosphorylated neurofilament protein, CD34, CD31, HMB-45, S-100, leukocyte common antigen, and BAF47/INI-1 (retained nuclear positivity). The Ki67 proliferative index was 9%. A diagnosis of desmoplastic small round cell tumor (DSRCT) was favored initially.^7^ Because *EWSR1* breakapart fluorescence in situ hybridization confirmed an *EWSR* rearrangement but concomitant *WT1* breakapart fluorescence in situ hybridization was negative, the molecular criterion for DSRCT was not met, and a final diagnosis of poorly differentiated sarcoma, not otherwise specified, was rendered. The patient received six cycles of induction chemotherapy—ifosfamide, carboplatin, and etoposide—followed by autologous stem-cell transplantation with a high-dose preparative regimen of carboplatin, thiotepa, and etoposide as well as 54 Gy of focal radiation to the location of the original tumor. After a 2-year remission, the tumor recurred with numerous pulmonary lesions in all lobes. The histologic characteristics of the metastasis were identical to the primary tumor. The patient enrolled in the Personalized OncoGenomics (POG)^3^ study, which offers whole-genome sequencing (WGS) and transcriptome sequencing and analysis to identify drivers and potential therapeutic options of relapsed solid tumors for children and adults in British Columbia.

**Fig A1.**
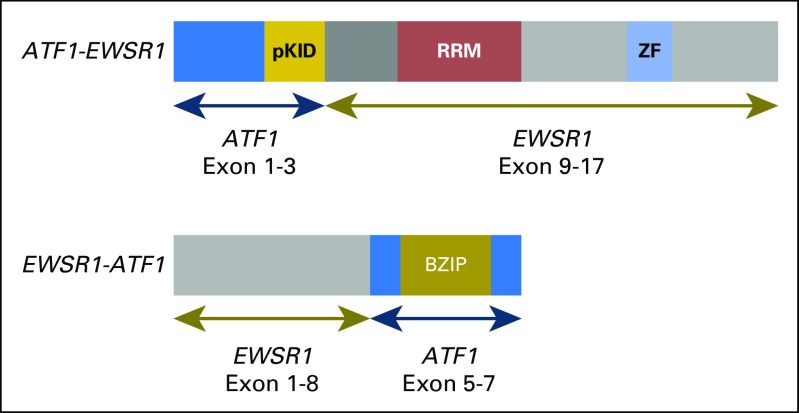
Schematic representation of the *EWSR1*-*ATF1* fusion. Two forms of the fusion were identified; the *EWSR1*-*ATF1* form had higher expression than the reciprocal *ATF1*-*EWSR1* form. BZIP, basic leucine zipper domain, which mediates sequence specific DNA binding properties and the leucine zipper that is required to hold together (dimerize) two DNA binding regions; pKID, phosphorylated kinase-inducible-domain; RRM, RNA recognition motif; ZF, Zn-finger in Ran binding protein and others.

Biopsy material from a lung metastasis was characterized with WGS and RNA-Seq, and peripheral blood was characterized with WGS.^3^ The analysis of the sequencing data revealed an *EWSR1-ATF1* gene fusion (Appendix Fig A1, online only); although this finding is most suggestive of a clear cell sarcoma, no immunohistochemical support for this diagnosis could be established.^8,9^ The POG team identified three somatic variants of unclear therapeutic significance: *PDGFRA* p.V299F, *PRKCB* p.D341N and *SVIL* p.L1374R. No germline single-nucleotide variants with established cancer relevance were detected.^10^ Although no therapy was available to target the EWSR1-ATF1 fusion protein directly, the POG RNA-Seq–derived gene expression analysis identified high expression of downstream genes *IL6* and *JAK1*. The finding of the *JAK1* overexpression was corroborated by comparative RNA-Seq analysis at the University of California Santa Cruz.

## COMPARATIVE RNA-SEQ ANALYSIS

**Fig 2.**
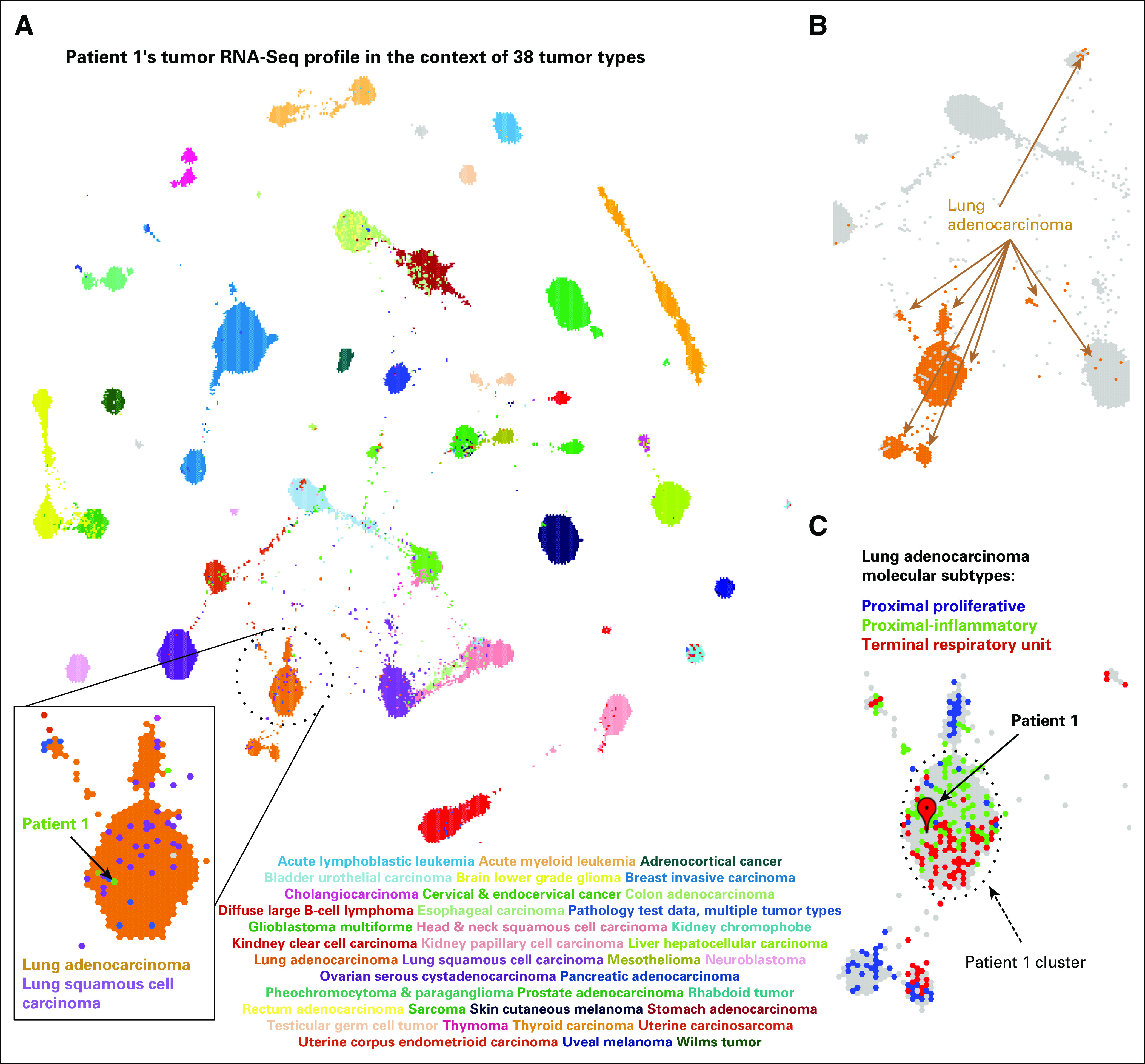
RNA-Seq–based gene expression profile for patient 1 visualized in the context of the reference cohort of 38 adult and pediatric tumor types. (A) A projection of the entire tumor cohort in two dimensions according to the TumorMap method. Individual tumors are represented by hexagons, and colored tumors by the tumor type, as indicated in the graphic. The tumor in patient 1 is shown in green within the lung tumors. (B) Lung adenocarcinoma (LUAD) tumors are found in four main regions of the TumorMap visualization. LUAD tumors are depicted in orange, whereas all other tumor types are in gray. (C) A zoomed-in view of the cluster for patient 1 and the surrounding area that contains LUAD tumors, now colored according to LUAD molecular subtypes (proximal proliferative, blue; proximal inflammatory, green; terminal repiratory unit, red). Unclassified samples are colored in gray.

**Fig A2.**
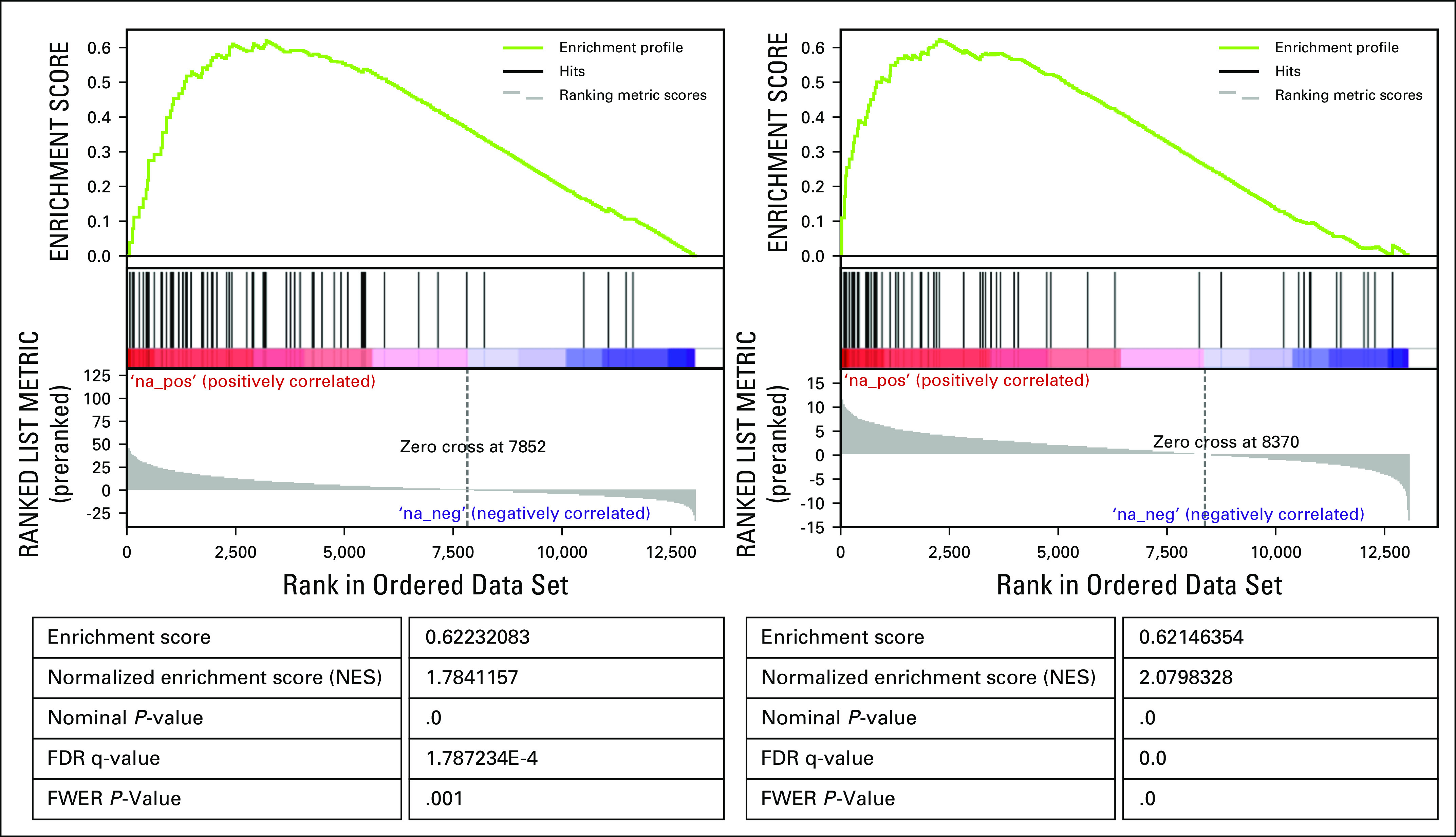
Relative expression levels of *EWSR1*, *ATF1*, and *JAK1*, compared to different cohorts of adult (TCGA) and pediatric (TARGET) tumours. Patient 1 tumor gene expression levels are indicated by the red vertical line. The two cohorts used for the outlier analysis are highlighted in gold. Ped, pediatric tumor types; TARGET, Therapeutically Actionable Research to Generate Effective Treatments; TGCA, The Cancer Genome Atlas.

In accordance with the US Food and Drug Administration guidelines,^6^ we focused on relative rather than absolute gene expression levels and sought to develop a framework for the analysis of RNA-Seq data from patients. We compared the RNA-Seq–derived tumor gene expression profile of patient 1 with similarly derived profiles of 10,668 samples that represented 38 pediatric and adult tumor types studied by the The Cancer Genome Atlas (TCGA) and Therapeutically Actionable Research to Generate Effective Treatments (TARGET).^11,12^ RNA-Seq reads from different laboratories were reanalyzed with a single computational pipeline to reduce batch effects.^13^ We searched for tumors in this homogeneously processed compendium in which expression profiles were similar to those of patient 1 by using TumorMap.^14^ The tumor gene expression profile for patient 1 resembled lung cancers (Fig 2A), the site of the metastasis. The metastatic sample contained 76% tumor cells, estimated by a POG pathologist, which suggested that most of the gene expression information came from the tumor cells. Lung adenocarcinoma (LUAD) samples formed four groups in the TumorMap (Fig 2B) and the sarcoma of patient 1 clustered with the 354 LUAD tumors of the terminal respiratory unit and proximal-inflammatory molecular subtype (Fig 2C), associated with the activation of receptor tyrosine kinases (RTK).^15^ To define the transcriptional programs that drove placement of the patient’s tumor with the lung cancers, we conducted Gene Set Enrichment Analysis^16^ with genes differentially expressed between the LUAD cluster that contained the tumor of patient 1 (n = 354) and the remaining samples in the compendium (n = 10,314); we also repeated this analysis and compared the cluster for patient 1 with the remaining LUAD samples (n = 529). Both analyses revealed the overexpression of members of the IL6/JAK/STAT3 signaling pathway (Appendix Fig A2), which suggests that the activation of shared signaling programs likely contributed to the tumor transcriptional phenotype of patient 1 in addition to the site of the metastatic sample. We next searched for genes that were significantly overexpressed in the patient’s tumor compared with the whole compendium and compared with only the sarcomas by using outlier statistics^3,17^ (Data Supplement). To explicitly subtract the effect of the lung cell expression, we also searched for outlier genes compared with 529 LUAD tumors; 787 genes, including druggable targets *JAK1*, *ALK*, *NTRK1*, and *CCND1*, emerged as overexpression outliers in all analyses (Data Supplement).

## MOLECULAR RATIONALE FOR CLINICAL DECISION MAKING

**Fig 3.**
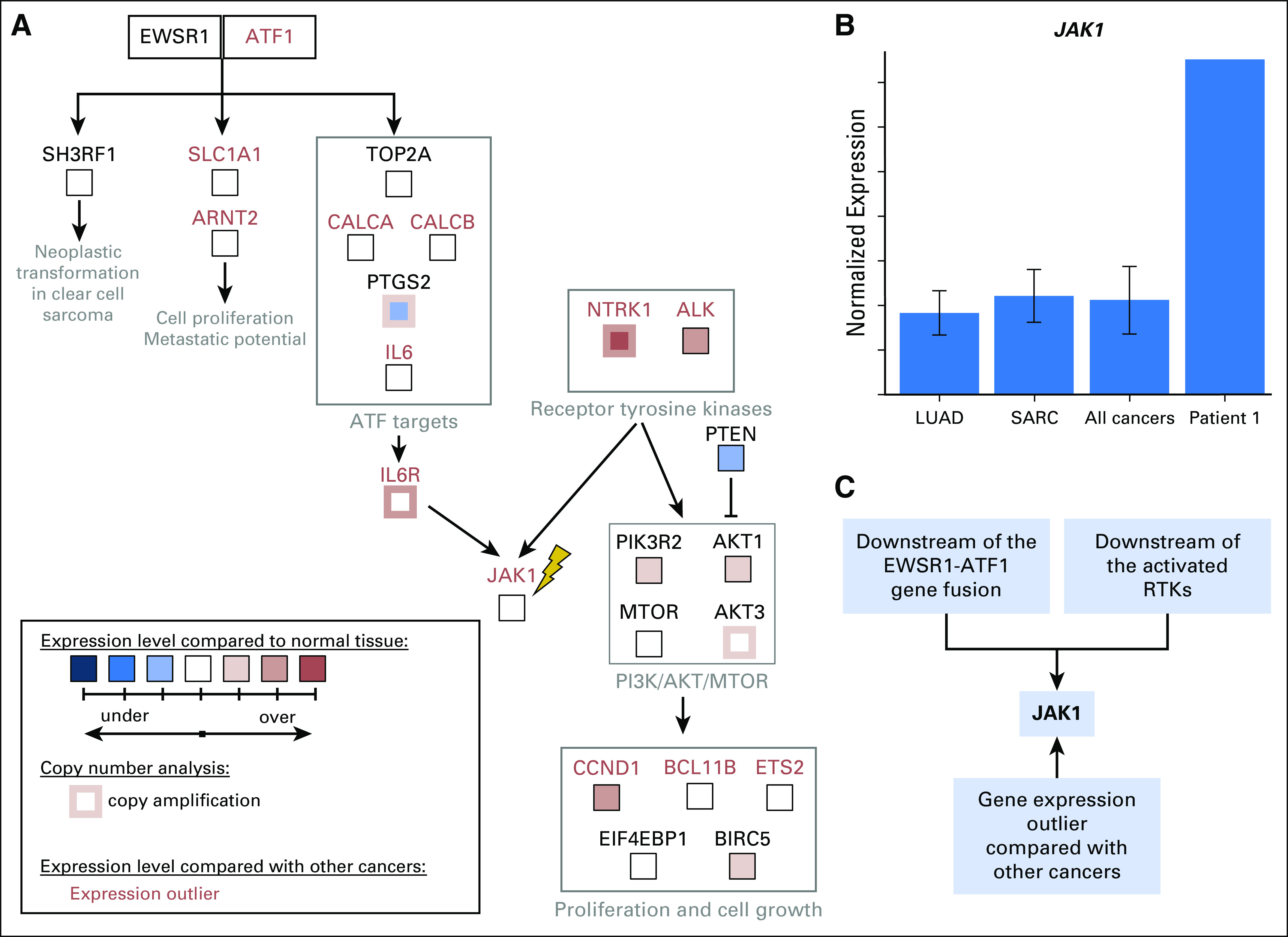
Molecular rationale for using ruxolitinib to treat the sarcoma in patient 1. (A) Candidate pathway that drove tumorigenesis in patient 1 was reconstructed on the basis of outlier analysis, differential expression analysis compared with normal tissues, copy number information, and literature mining (Appendix Methods). Both EWSR1-ATF1 and receptor tyrosine kinases NTRK1 and ALK can contribute to the activation of IL6/JAK/STAT3 signaling. All gene expression outliers depicted in the figure (gene names written in red font) were significant in all three comparisons: patient 1 versus all cancers, patient 1 versus lung adenocarcinomas, and patient 1 versus sarcomas. JAK1, the molecular target of ruxolitinib, is indicated with a yellow lightning bolt. (B) The tumor in patient 1 expresses *JAK1* at a strikingly higher level than those seen in all 10,668 tumors, which are represented by 38 tumor types studied by the TCGA^11^ and TARGET (denoted PANCAN),^12^ including lung adenocarcinomas (LUADs) and sarcomas (SARCs). (C) JAK1 is an attractive molecular target for patient 1’s tumor because it is downstream of the EWSR1-ATF1 fusion and the activate receptor tyrosine kinases (RTKs). It was also identified as over expressed by gene expression outlier analysis.

**Fig A3.**
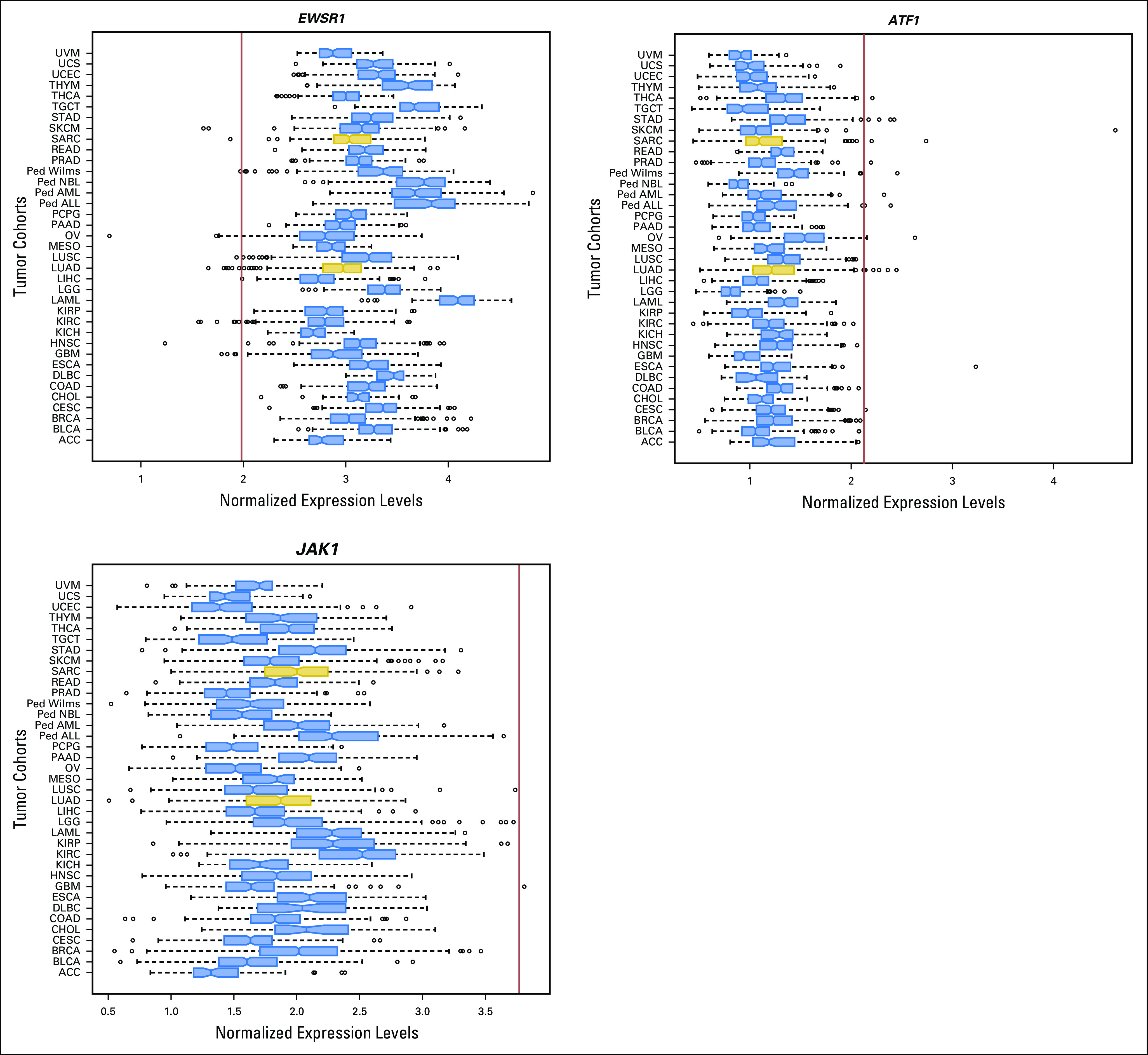
Gene Set Enrichment Analysis (GSEA) of the gene scores obtained through differential gene expression analysis comparing patient 1’s TumorMap cluster to the whole reference compendium (left) and to the remaining TCGA LUAD samples (right) identifies IL6/JAK/STAT3 pathway as one of the most significantly enriched pathways in genes differentially overexpressed in patient 1’s TumorMap cluster.

**Fig A4.**
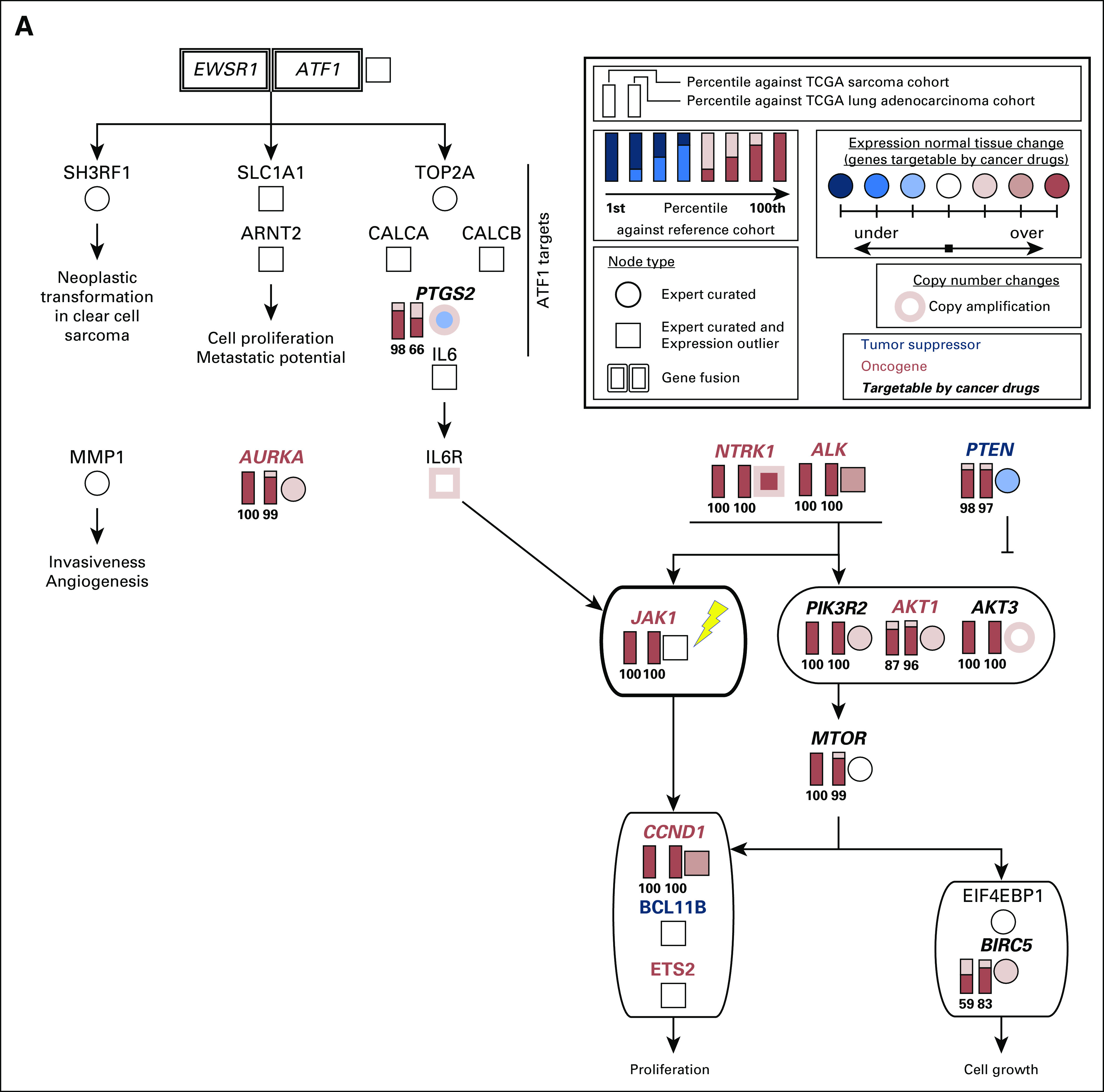
Full candidate pathway that represents molecular drivers of tumorigenesis in the sarcoma of patient 1. We reconstructed this pathway on the basis of the outlier analysis, differential gene expression analysis, copy number information and literature mining (Appendix Methods). A simplified version of this pathway is presented in Figure 3A. Both EWSR1-ATF1 and receptor tyrosine kinases PDGFRB, NTRK1, ALK, and FGFR1 can contribute to the activation of IL6/JAK/STAT3 signaling. All gene expression outliers depicted in this figure were significant in all three comparisons: patient 1 versus all cancers, patient 1 versus lung adenocarcinomas, and patient 1 versus sarcomas. JAK1, the molecular target of ruxolitinib, is indicated with a yellow lightning bolt. TCGA, The Cancer Genome Atlas.

We speculated that the activation of RTKs contributed to JAK over-expression in patient 1’s tumor.^18,19^ Increased expression of *ATF1* and its transcriptional targets, *TOP2A*, *CALCA*, and *IL6*, was observed, presumably as a result of constitutive transcriptional activation by EWSR1-ATF1 (Fig 3A; Appendix Fig A3). ATF1 can activate the transcription of *JAK1*,^8^ providing another potential mechanism for the observed high expression of the IL6/JAK/STAT3 pathway. Consolidating the fusion-based and the RTK-based mechanisms of IL6/JAK/STAT3 activation, we reconstructed a candidate pathway driving patient 1’s cancer (Fig 3, Appendix Fig A4). The POG molecular tumor board suggested targeting either JAK (with ruxolitinib) or ALK (with crizotinib). A decision was made to use ruxolitinib given (1) the over-expression of *ATF1* target genes, (2) the over-expression of *JAK1*, and (3) the available pediatric dosing information.^20^ In addition, ruxolitinib was favored over crizotinib because it targets downstream of both EWSR1-ATF1 and the over-expressed RTKs, whereas crizotinib only targets the RTKs. We recognize that a combination therapy targeting both ALK and JAK may have been appropriate on the basis of the molecular findings. However, we were unable to use drug combinations that have not been through phase 1 testing, highlighting the need for more pediatric combination therapy trials.

## CLINICAL RESPONSE

**Fig 4.**
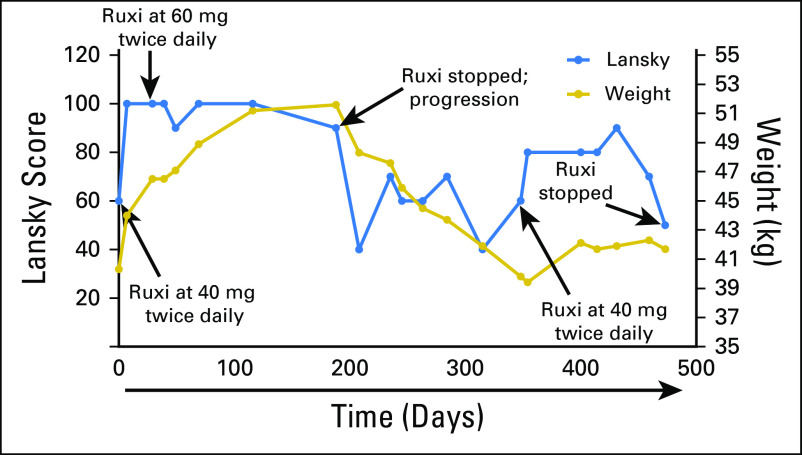
Clinical response of patient 1 to ruxolitinib (Ruxi). Ruxi was initiated at day 0 at 40 mg twice per day. The patient’s body weight was 40.3 kg, and the Lansky play-performance score was 60 at the time of treatment initiation. During treatment, the patient’s status improved significantly according to both body weight and Lansky play-performance score, which reached normal levels; thus, the Ruxi dose was increased to 60 mg twice per day at day 29. The patient continued to receive this dose until disease progression according to computed tomography occurred at day 188 (Ruxi stopped; progression). Ruxi was restarted at day 348, and a response again was noted according to both body weight and Lansky performance score.

At the initiation of therapy with single-agent ruxolitinib, patient 1 had severe nausea and lethargy, was mostly bed-ridden, and had a Lansky play-performance score^21^ of 60 (Fig 4). Within a week of ruxolitinib initiation, his mother reported a dramatic improvement in his energy level and complete resolution of his nausea. The patient tolerated this therapy without significant toxicity and had stabilization of the previously rapidly growing lung nodules by RECIST,^22^ and his Lansky score improved to 90 to 100 for 5 months. The patient then exhibited a sudden enlargement in one lung lesion, detected during routine imaging, although most of the other lesions remained stable. Ruxolitinib was discontinued, and focal palliative radiation was administered to the one lesion in the left lower lobe for pain control. Within 2 months of ruxolitinib discontinuation, the symptoms of nausea, extreme fatigue, and weight loss returned, and the lung lesions progressed. The family requested that ruxolitinib be restarted for quality of life, and the patient again showed dramatic improvement in clinical status and an unexpected prolonged period of stable disease until dose reduction because of myelosuppression was required. After the dose reduction, rapid progression of the pulmonary lesions resulted in death 23 months after the original relapse.

## DISCUSSION

To our knowledge, this is the first report of a pediatric patient with cancer who benefited from cross-tumor gene expression comparisons. Cross-tumor analyses have been used in the TCGA^11^ and POG studies^3,23,24^; however, a computational framework is necessary for their clinical implementation. This case is also, to our knowledge, the first report of use of a *JAK* inhibitor to treat a sarcoma. Previous functional studies implicated *STAT3* as an oncogene in sarcomas^25^; the current case report builds on this work and prompts investigation into the potential clinical utility of targeting this pathway. Of note was the patient’s marked and rapid clinical response to treatment, which suggests that response may have been related to the modulation of cytokine expression by the medication. Although the clinical benefit of ruxolitinib was apparent, it was challenging to quantify. Ultimately, a randomized clinical trial is necessary to assess the benefit of molecular approaches compared with the standard of care.

The case also highlights tumor heterogeneity: although the majority of the metastases remained stable, one lesion rapidly became resistant. Despite documented progressive disease, this case benefited from resumption of the medication: the patient’s clinical status and many metastatic lesions were responsive to retreatment. A serial molecular analysis of the heterogeneous lesions could inform the mechanisms of resistance; however, it was not pursued because of the family’s wishes. To characterize the intratumor heterogeneity of therapeutic response, we consider follow-up biopsies, and those decisions are weighed against the risks to the patient.

## Figures and Tables

**Table A1.**
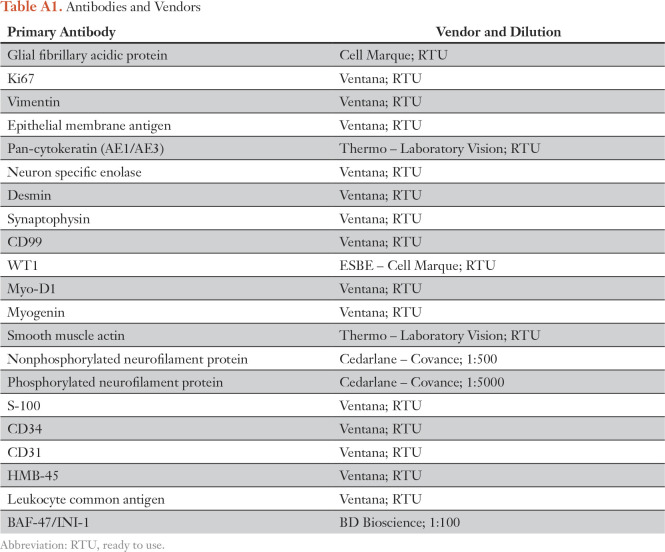
Antibodies and Vendors

## Appendix

### Online Methods

#### Reference compendium

We obtained The Cancer Genome Atlas (TCGA)^11^ and Therapeutically Applicable Research to Generate Effective Treatments (TARGET)^12^ RNA sequencing fragments per kilobase of transcript per million mapped reads (FPKM^12^; Trapnell C, et al: Natl Biotechnol 28:511-515, 2010) gene expression data from the public data hub in the University of California Santa Cruz Xena Browser (Vivian J, et al: Nat Biotechnol 35:314-316, 2017; Goldman M, et al: Nucleic Acids Res 43:D812-D817, 2015). The TCGA and TARGET data sets were processed with the same RNA-Seq pipeline (STAR2 [Dobin A, et al: Bioinformatics 29:15-21, 2013] and RSEM [Li B, et al: BMC Bioinformatics 12:323, 2011]) by the University of California Santa Cruz Genomics Institute.^13^ We extracted tumor samples from these data sets and combined them into a single cohort that contained multiple adult and pediatric tumor types (N = 10,668). The expression of 18,357 protein-coding genes was measured (Data Supplement).

#### Gene expression outlier analysis

Gene-level reads per kilobase of transcript per million mapped reads (RPKM) expression measurements for patient 1's tumor were generated according to the previously published method.^3^ We used these data to compute FPKMs by dividing each value by two. We proceeded to quantile normalize the expression values, using theoretical exponential distribution (rate parameter = 1) as a background distribution (Bolstad BM, et al: Bioinformatics 19:185-193, 2003). During this procedure, we normalized expression quantiles within each tumor sample to match quantiles of an exponential distribution (rate parameter = 1). We then performed gene expression outlier analysis^3^^,^^17^ to identify transcripts significantly enriched in the patient's tumor compared with 10,668 cancer samples in the reference compendium (pan-cancer outlier analysis). We also performed the same analysis using only the 529 lung adenocarcinoma (LUAD) tumors as a reference (LUAD-only outlier analysis) and using 262 sarcoma tumors as a reference (sarcoma-only outlier analysis). Gene expression outliers were identified as described,^17^ with the exception of the use of a more stringent interquartile range of 2.0. We analyzed the outlier genes for enrichment of specific pathways and signaling networks that could be targeted by available therapies using MSigDB (Liberzon A et al: Bioinformatics 27:1739-1740, 2011).

The pan-cancer outlier analysis of the tumor in patient 1 compared with the reference compendium (n = 10,668) revealed 906 genes significantly overexpressed in the patient’s tumor. The LUAD-only outlier analysis^3^^,^^17^ (n = 529) revealed 1,176 up-outlier genes, and the same analysis against sarcomas (n = 262) revealed 1,196 genes. Seven hundred eighty-seven genes were identified as up-outliers by all three analyses. We focused our analysis on the up-outlier genes, because abnormally activated genes represent the most tractable therapeutic targets. We used the Drug Gene Interaction Database (DGIdb; Wagner AH, et al: Nucleic Acids Res 44:D1036-D1044, 2016) to identify genes whose protein products could be targeted by clinically available inhibitors. The outlier analysis results are listed in the Data Supplement.

### TumorMap Analysis

TumorMap^14^ (https://tumormap.ucsc.edu/) is a genomic portal browser that allows visualization and navigation of a high-dimensional genomic space in a two-dimensional Euclidean projection, in a manner similar to geospatial two-dimensional maps. In this projected space, samples are laid out on the basis of similarities of their RNA-Seq–based gene expression profiles. Samples that cluster together exhibit similar gene expression profiles.

We first computed pairwise Spearman correlations (Brown GW: Arch Pediatr Adolesc Med 146:682, 1992) between RNA-Seq–derived gene expression profiles of all tumor pairs in our reference cohort (n = 10,668), including the tumor in patient 1 (N = 10,669). This produced a square correlation matrix with 10,669 columns and 10,669 rows.

The TumorMap method seeks to project high-dimensional genomic observations onto a two-dimensional plane while preserving original sample-to-sample distances. Tumors cluster together according to the similarity of their RNA-Seq–derived gene expression profiles. We used the quasi-physics–based layout engine OpenOrd (formerly known as DrL; Martin et al: Proc SPIE. 786806, 2011), implemented in the igraph R package (Gabor C, et al: InterJournal Complex Systems 1695, 2006), to derive an initial set of (x, y) positions for the samples on the basis of the correlation matrix (Wylie BN et al: Visualization of Information Spaces with VxInsight, Sandia National Labs SAND2000-3100, 2000). The similarity space is represented as a graph and is used as an input into OpenOrd. OpenOrd treats the similarities as spring constants and searches for a configuration among the samples that produces an arrangement to minimize the spring tension of the system as much as possible. We use hexagonal packing for space conservation in the projected two-dimensional plane. For each sample in the full correlation matrix, we extracted samples with top six correlation values to compose a sparse matrix of the top six nearest neighbors. We used this sparse matrix to construct a sparse similarity graph for the samples in the cohort and applied the OpenOrd method to derive the initial (x, y) positions in the map.

Furthermore, to avoid overlapping and crowding samples in the dense graph components, OpenOrd (x, y) coordinates are snapped to their nearest hexagon to arrange all of the samples on a tiling of regular hexagons. With OpenOrd (x, y) coordinates, each sample is placed in a grid cell. If the predetermined cell is occupied, the sample is snapped to an empty grid cell within a minimal distance from the original cell. Multiple samples that compete for a location will thus spiral around a central hexagon in the neighbors around the central location. Therefore, dense clumps are separated so that they can be viewed on approximately the same scale as the distances that separate them. Hexagons were selected as the shape for the grid cell to illustrate that there are no inherently preferred axis-aligned directions in the OpenOrd output.

Google Maps Application Programming Interface (API; https://developers.google.com/maps/documentation/javascript/reference) is used to load and visualize the resulting layout in a browsing environment. The API provides the ability to interactively navigate, zoom, and explore various annotations of locations on the map, analogous to Google Maps and Google Earth applications.

We applied the TumorMap method to the reference cohort of 10,668 tumors together with the tumor of patient 1 by using transcriptional profiles of 18,357 genes (Data Supplement). Of note, the reference compendium contained 262 heterogeneous sarcomas from the TCGA^11^ cohort.

#### Statistical Robustness of TumorMap Placement

The TumorMap method belongs to the family of nearest neighbor classification methods. It projects the similarity space of the high-dimensional genomic profiles into Euclidean space by using only top neighbors of every sample in the cohort. We refer to these top neighbors as the local neighborhood of a given sample. Therefore, it is important to evaluate how robust these local neighborhoods are under small perturbations. Specifically, we wanted to assess whether the local neighborhood of patient 1 remains stable when only subsets of genes are used to compute pairwise similarities between samples.

We subsampled, without replacement, gene expression features at 80% of the original gene features. We repeated this procedure 1,000 times and computed the patient’s local neighborhood across all N = 1,000 subsampled spaces. We then compared each of these local neighborhoods computed under perturbation to the true local neighborhood computed with the complete data set. We computed a local neighborhood specificity (LNspecificity) score as follows:

$$LNspecificity\;=\;\frac{1}{N}*\sum_{i=1}^{N=1000}\frac{S^{true}\cap \;S^{i}}{\left|S^{i}\right|},$$

where S is a set of nearest neighbors for either true or subsampled computation and $\left|S\right|$is the nearest neighbor set cardinality.

This score represents average overlap, as a fraction, of the true local neighborhood and the perturbed local neighborhoods across all subsampling iterations. The higher this score is, the more overlap we see across all the iterations and the more similarities we see among local neighborhoods under perturbations. The SP score of patient 1 was 0.885, which indicated that 88.5% of the top neighbors were consistently the same across 1,000 perturbations to the gene expression profiles, from which the TumorMap visualization was computed.

### Identification of Genes Associated With the Patient 1 Cluster in TumorMap

We identified genes differentially expressed between the LUAD cluster containing patient 1 and the 10,668 tumor samples in the compendium by using the Linear Model for Microarray Analysis method (Ritchie ME, et al: Mucleic Acids Res 43:e47, 2015). The gene set enrichment analysis (GSEA)^16^ of the resulting list of genes revealed that IL6/JAK/STAT3 signaling pathway annotation was significantly enriched among these genes (false discovery rate q value, 1.787^e−4^ with 39 genes in the leading edge).

We also identified differentially expressed genes between the LUAD cluster containing patient 1 and the remaining LUAD tumors in the compendium (Data Supplement). The GSEA analysis^16^ of this gene list revealed that members of the IL6/JAK/STAT3 signaling pathway were still driving the creation of the LUAD cluster containing patient 1's tumor (false discovery rate q value, 0 with 36 genes in the leading edge).

### Identification of Druggable Targets

We used the DGIdb (Liberzon A et al: Bioinformatics 27:1739-1740, 2011; Wagner AH, et al: Nucleic Acids Res 44:D1036-D1044, 2016) resource to search for drug targets among the genes that were upregulated in patient 1 by gene expression outlier analysis. We chose four databases—MyCancerGenome, MyCancerGenome Clinical Trial, CIVIC, and Cancer Commons—to focus on drug targets with known cancer relevance. We chose the following six interaction types: antagonist, antibody, blocker, inhibitor, inhibitory allosteric modulator, and suppressor.

### Gene Expression Percentile Analysis

For the druggable targets in the reconstructed candidate driver pathway , we computed the ranked percentiles of the gene expression levels in patient 1's tumor compared with the TCGA LUAD (n = 529) and TCGA SARC (n = 264) cohorts. For each sample in the cohort as well as for patient 1, we ranked genes relative to the expression of all genes within that sample (ie, the highest expressed gene was ranked as 1). The percentiles are displayed in Figure 3 for 12 genes that can be targeted by known cancer drugs, according to DGIdb analysis (Liberzon A et al: Bioinformatics 27:1739-1740, 2011; Wagner AH, et al: Nucleic Acids Res 44:D1036-D1044, 2016).

### Differential Expression Analysis Relative to Normal Tissues

For all of the genes in the reconstructed candidate driver pathway that are targetable by cancer drugs (Fig 3) we computed the fold change between the expression in patient 1's tumor and in normal cells. We obtained normal tissue expression data for 16 different tissues from the Illumina Human Body Map 2.0 database (GEO Accession No. GSE30611). For each gene, we mean-aggregated normal expression into a single value and computed log change of the expression of that gene in patient 1 compared with the aggregated normal value.

### Histologic Techniques and Immunohistochemistry

The tumor tissue was routinely fixed in 10% buffered formalin and then was processed in an automated fashion. Processed tissue was then embedded in paraffin wax, and the resultant blocks were sectioned at 4 µm for both hematoxylin and eosin staining and immunohistochemistry. All microscopic slides were prepared via standard automated techniques. Immunohistochemical slides were stained on the Ventana BenchMark XT Autostainer (Ventana Medical Systems, Tucson, AZ) with the Ventana iVIEW universal DAB detection kit. Primary antibodies used for immunohistochemistry are noted in Table A1 and the Data Supplement. All primary antibodies were either diluted or received as a ready to use prediluted solution from the relevant vendor.

### Fluorescence In Situ Hybridization

Fluorescence in situ hybridization analysis was performed on formalin-fixed paraffin-embedded tumor tissue. Overall, 200 nuclei were quantified with the *EWSR1* (22q12) dual-color breakapart probe (Vysis; Downers Grove, IL). Likewise, 200 nuclei were quantified with the *WT1* (11p13) dual-color breakapart probe (prepared at the BC Cancer Agency). Standard, internally derived thresholds were followed to determine definitive presence of a translocation that involved the *EWSR1* and *WT1* loci.

### Additional Information

The Personalized OncoGenomics study was approved by the University of British Columbia Research Ethics Committee (No. H12-00137), and written informed consent was obtained from the family of patient 1 before genomic profiling occurred. The Treehouse Childhood Cancer Initiative was approved by the institutional review board at the University of California Santa Cruz (No. HS2648). Patient identity was anonymized within the research teams, and an identification code was assigned to the case to communicate clinically relevant information to physicians. The family consented to potential publication of findings. Raw sequence data and downstream analytics were maintained within a secure computing environment.

Treehouse Childhood Cancer Initiative (https://treehousegenomics.soe.ucsc.edu), a partnership between the University of California Santa Cruz Genomics Institute and pediatric oncology centers, is designed to systematize RNA-Seq comparisons. We note that these comparisons become more effective when molecular and clinical information is shared, which ensures that each new case is informed by all relevant previous cases. We hope that Treehouse will streamline data sharing and comparative RNA-Seq analysis so that they can occur in real time.
